# A Case of Herpes Simplex Virus Colitis in an Immunosuppressed Patient

**DOI:** 10.7759/cureus.51409

**Published:** 2023-12-31

**Authors:** Jasmine Tidwell, Minh Thu T Nguyen, Faripour Forouhar, Campbell L Stewart, Roopjeet Bath

**Affiliations:** 1 Internal Medicine, University of Connecticut Health, Hartford, USA; 2 Gastroenterology and Hepatology, University of Connecticut, Farmington, USA; 3 Pathology, University of Connecticut Health, Farmington, USA; 4 Gastroenterology and Hepatology, University of Connecticut Health, Farmington, USA

**Keywords:** systemic lupus erythematous, acyclovir, immunosuppression, colitis, herpes simplex virus

## Abstract

Herpes simplex virus (HSV) can cause severe disseminated infections in immunocompromised patients. Gastrointestinal tract involvement seldom includes the colon. We present a rare case of disseminated cutaneous HSV infection with concomitant colonic involvement in an immunosuppressed patient. The patient’s clinical presentation and computerized tomography (CT) findings were concerning for colitis. She failed to improve on antibiotic therapy and subsequently underwent flexible sigmoidoscopy. Gross findings and histopathology were consistent with herpes simplex virus colitis. It is essential to recognize this pathology in immunocompromised patients to evaluate the need to hold immunosuppressive therapy and ensure successful treatment to prevent fatal outcomes.

## Introduction

Worldwide, herpes simplex virus (HSV) types 1 and 2 are among the most widespread viruses, with a prevalence of 67% and 13%, respectively [1]. They cause a variety of pathologies, ranging from cold sores to colitis [2]. The virus penetrates the skin and then mobilizes to the peripheral neurons, remaining latent until reactivation [3]. The risk of reactivation increases with immunosuppressive therapy, which may lead to severe and disseminated infections [4]. Gastrointestinal tract involvement typically includes the esophagus, perineum, or rectum [5]. We describe a rare case of disseminated cutaneous HSV infection with concomitant colonic involvement in an immunosuppressed patient.

This case was previously presented as an abstract at the 2023 ACG (American College of Gastroenterology) meeting on October 24, 2023.

## Case presentation

**Figure 1:**
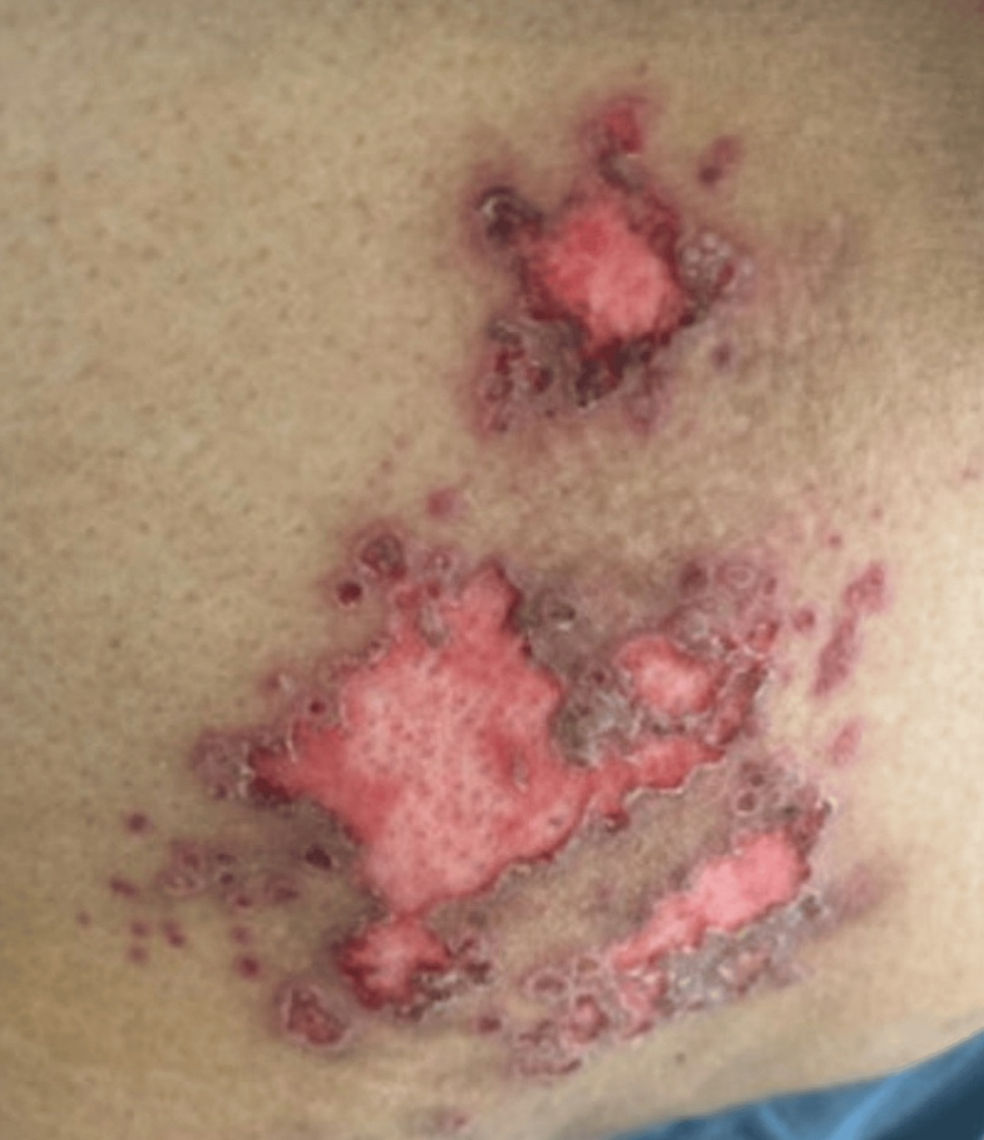
Left buttock with a disseminated viral rash

A 48-year-old woman with a past medical history of systemic lupus erythematosus (SLE) presented due to a rash. Her home medications included mycophenolate mofetil (MMF) 1 gram twice daily, voclosporin 23.7 milligrams (mg) twice daily, and prednisone 20 mg daily. The rash developed 10 days prior, starting on her left buttock (Figure 1).

She described it as vesicular, superficial, itchy, and painful with associated fevers and chills. She was evaluated in a rheumatology clinic with concern for eczema, for which she was prescribed topical hydrocortisone. However, the rash failed to improve, began oozing serous discharge, and became more painful. It rapidly spread to the labia majora and perianal region, for which she presented to the emergency department.

**Figure 2:**
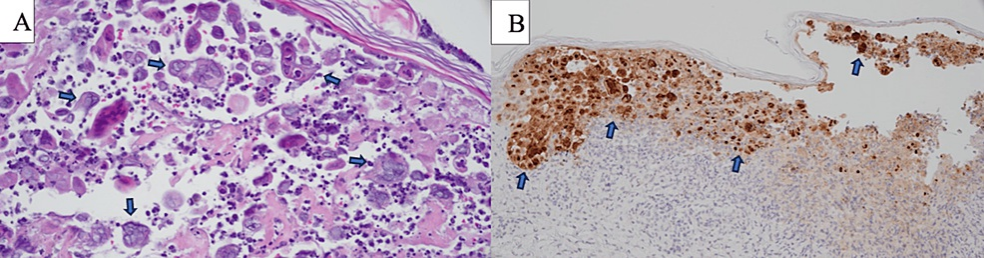
Punch biopsy of the left buttock (A) Herpes simplex virus (skin, left buttock, H&E, 60 x): The keratinocytes demonstrate multinucleation, margination of the chromatin, and nuclear molding. Necrotic and acantholytic keratinocytes are surrounded by primarily neutrophilic inflammation. (B) Herpes simplex virus (skin, left buttock, HSV immunohistochemistry, 20X): The nuclei of the keratinocytes are positive for herpes simplex stain (Cell Marque, Rocklin, CA).

Upon arrival, she was hemodynamically stable. Labs were remarkable for neutrophilic leukocytosis, with a white blood cell count of 16.8 grams per deciliter with 92.5% neutrophils. She was admitted for further workup and treatment of the rash. Skin viral polymerase chain reaction (PCR) assay was positive for herpes simplex virus type 2. A punch biopsy of her left buttock showed intraepidermal vesiculation, epidermal necrosis, epidermal clefting, and acantholysis. The keratinocytes showed multinucleation, margination of the chromatin, and nuclear molding with a predominantly neutrophilic dermal infiltrate extruding into the necrotic epidermis (Figure 2A). Immunohistochemistry was positive for HSV (Figure 2B).

**Figure 3:**
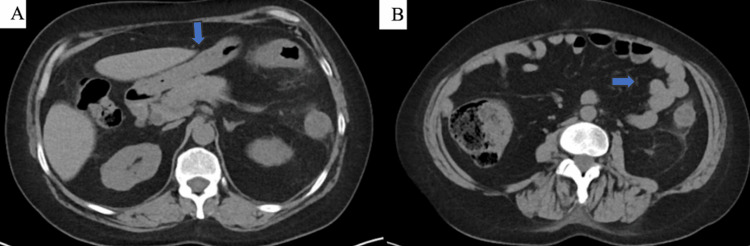
CT abdomen and pelvis with IV and oral contrast Colonic wall thickening and stranding involving the transverse colon (A) and descending colon (B).

Her hospital course was complicated by an average of 12 episodes of watery diarrhea daily with hematochezia and small blood clots. She also reported decreased appetite and left flank pain. She denied nausea, vomiting, or previous rectal bleeding. Her hemoglobin and hematocrit remained stable despite the bloody bowel movements. Computerized tomography (CT) scan of the abdomen and pelvis with intravenous (IV) and oral contrast revealed colonic wall thickening and stranding involving the transverse colon and descending colon (Figures 3A, 3B).

She was empirically treated with levofloxacin IV 750 mg daily for three days for presumed colitis. Stool cultures, polymerase chain reaction (PCR) panels, and ova and parasites resulted negative. 

**Figure 4:**
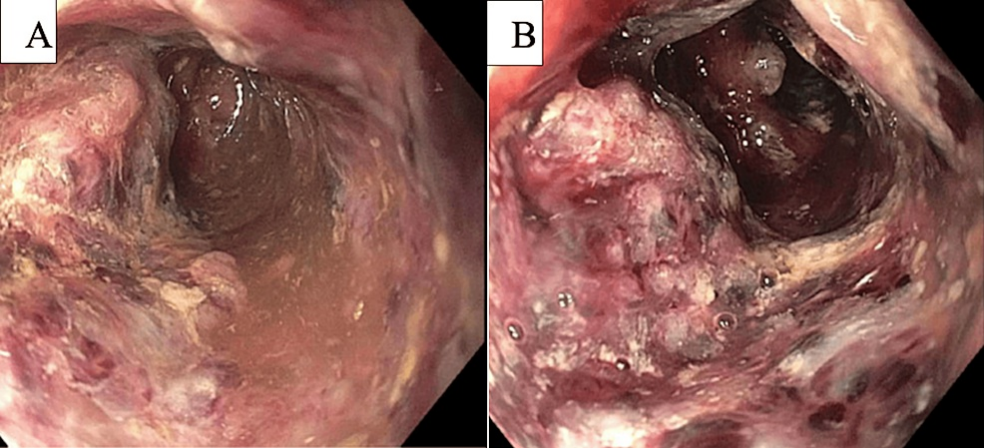
Flexible sigmoidoscopy Deep ulcers and necrotic mucosa in the descending colon (A) and splenic flexure (B).

Due to continued diarrhea and abdominal pain despite antibiotic therapy, flexible sigmoidoscopy was performed for further evaluation. It showed discontinuous areas of ulcerated and necrotic mucosa with stigmata of recent bleeding in the rectum, sigmoid colon, descending colon, splenic flexure, and mid and distal transverse colons (Figures 4A, 4B).

**Figure 5:**
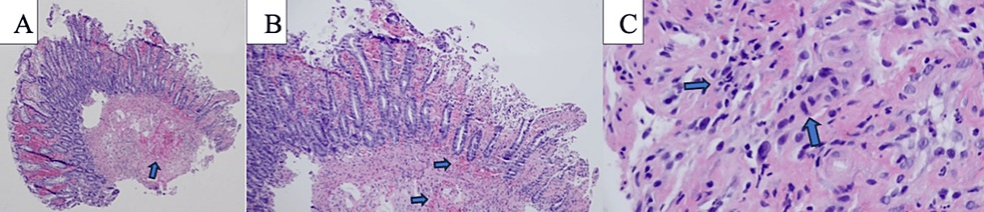
Colonic biopsy A (4X), B (100X), C (40x): Hematoxylin and eosin-stained sections showing multiple fragments of colonic mucosa with patchy hemorrhagic and ischemic changes. Also noted are scattered fibrinous and inflammatory exudate and small mononuclear aggregates.

Hematoxylin and eosin-stained sections contained multiple fragments of colonic mucosa with patchy hemorrhagic and ischemic changes. While some biopsy fragments appeared edematous but near normal, others had ischemic glandular attenuation, stromal hemorrhage, and surface ulceration with occasional pseudomembrane formation. Scattered fibrinous, inflammatory exudate and small mononuclear aggregates were also noted (Figures 5A-5C).

The pathologist noted that these changes were compatible with HSV colitis.

MMF and voclosporin were subsequently held. She received a 14-day course of IV acyclovir at 10 mg/kg every eight hours. Her diarrhea and abdominal pain improved, for which she was discharged. A week later, she followed up in the gastroenterology clinic and reported no further abdominal pain with normalized bowel movements.

## Discussion

HSV is commonly localized in the mucosa of the genitals, lips, or skin. However, immunosuppressed individuals can have severe disseminated infections, including colonic involvement, since cell-mediated immunity is the dominant process for controlling viral replication [6,7]. The clinical manifestations of HSV colitis can include fever, diarrhea, rectal bleeding, or abdominal pain [4]. Our patient presented with the cardinal symptoms of colitis.

The best method to diagnose HSV is via a PCR assay from the affected tissues or biopsies [7]. Herpetic colitis often involves the enterocytes but can extend to the submucosa [6,8]. As the virus penetrates the surface mucosa, it leads to cytolysis, causing a local inflammatory reaction, forming sharply defined, linear ulcers with intervening normal mucosa [6,8]. Pseudomembranous changes and aggregates of mononuclear cells can be present [6,8]. Histology of colonic biopsies often shows edema and patchy areas of ulceration; viral inclusions may be seen but are often rare or undetectable [6,8]. Diagnosis is typically made clinically and confirmed by compatible histology [6,8]. However, it is challenging to obtain invasive specimens from critically ill patients for which, in the correct clinical context, non-invasive tests can support the clinical diagnosis. In our case, cutaneous HSV was diagnosed via a PCR assay and confirmed by skin biopsy. HSV colitis was diagnosed based on the clinical presentation, gross findings on flexible sigmoidoscopy, and histopathology from colonic biopsies.

Our patient was treated with acyclovir, a nucleoside analog that selectively impedes the replication of HSV by inhibiting the viral deoxyribonucleic acid polymerase and is the first-line treatment [6]. In severe infections, IV therapy for two weeks is suggested [4]. Guidelines also recommend discontinuing immunosuppressant therapy in the setting of severe infections such as colitis [4,9]. In our case, MMF and voclosporin were initially held. Rheumatology encouraged continuing her daily prednisone for the management of underlying SLE. MMF was restarted four weeks after the resolution of symptoms, and voclosporin was discontinued permanently.

For prevention, there may be a role for prophylactic antiviral therapy in patients who are immunocompromised with serological evidence of latent HSV infections or a history of disseminated or tissue-invasive infections [10].

## Conclusions

We present a unique case of HSV colitis associated with disseminated cutaneous infection in an immunosuppressed patient. HSV colitis presents similar to other etiologies of colitis, including fever and rectal bleeding. Diagnosis is typically made clinically and confirmed by compatible histology, and management consists of antiviral therapy. The early recognition of HSV colitis in immunocompromised patients is crucial to reach a prompt diagnosis, evaluate the need to hold immunosuppression, and ensure successful treatment to prevent fatal outcomes.
